# Comprehensive Detection, Grading, and Growth Behavior Evaluation of Subthreshold and Low Intensity Photocoagulation Lesions by Optical Coherence Tomographic and Infrared Image Analysis

**DOI:** 10.1155/2014/492679

**Published:** 2014-05-12

**Authors:** Stefan Koinzer, Amke Caliebe, Lea Portz, Mark Saeger, Yoko Miura, Kerstin Schlott, Ralf Brinkmann, Johann Roider

**Affiliations:** ^1^Department of Ophthalmology, University of Kiel, House 25, Arnold-Heller-Straße 3, 24105 Kiel, Germany; ^2^Institute of Medical Informatics and Statistics, University of Kiel, House 31, Arnold-Heller-Straße 3, 24105 Kiel, Germany; ^3^Institute of Biomedical Optics, University of Lübeck, Peter-Monnik-Weg 4, 23562 Lübeck, Germany

## Abstract

*Purpose*. To correlate the long-term clinical effect of photocoagulation lesions after 6 months, as measured by their retinal damage size, to exposure parameters. We used optical coherence tomographic (OCT)-based lesion classes in order to detect and assess clinically invisible and mild lesions. *Methods*. In this prospective study, 488 photocoagulation lesions were imaged in 20 patients. We varied irradiation diameters (100/300 µm), exposure-times (20–200 ms), and power. Intensities were classified in OCT images after one hour, and we evaluated OCT and infrared (IR) images over six months after exposure. *Results*. For six consecutive OCT-based lesion classes, the following parameters increased with the class: ophthalmoscopic, OCT and IR visibility rate, fundus and OCT diameter, and IR area, but not irradiation power. OCT diameters correlated with exposure-time, irradiation diameter, and OCT class. OCT classes discriminated the largest bandwidth of OCT diameters. *Conclusion*. OCT classes represent objective and valid endpoints of photocoagulation intensity even for “subthreshold” intensities. They are suitable to calculate the treated retinal area. As the area is critical for treatment efficacy, OCT classes are useful to define treatment intensity, calculate necessary lesion numbers, and universally categorize lesions in clinical studies.

## 1. Introduction

Retinal photocoagulation is inexpensive and easy-to-administer, and the treatment requires only limited repetition and follow-up. It remains the basic therapy for peripheral retinal ischemia and an adjunctive therapy for diabetic macular edema [1]. On the other hand, it is a tissue destructive procedure [2]. Side-effects include acute pain, scotomas, reduced colour vision, decreased night vision, and uncontrolled atrophic scarring [3, 4]. Much effort has been undertaken to reduce lesion intensities [5–9], and pilot studies have collected evidence that “subthreshold” laser treatment can be effective [10–15].

Current concepts of “subthreshold” photocoagulation suffer from dissatisfactory endpoint definitions and from poorly reproducible lesion evaluations. Previous studies have used a variety of criteria to define lesion intensity, such as ophthalmoscopic invisibility [16], fluorescein angiographic (FLA) leakage [6, 11], optoacoustics [17], power titration according to reference lesions [18–20], long-term autofluorescence (AF) imaging [16], and others. We believe that modern optical coherence tomography (OCT) has the capacity to improve lesion definition significantly, as it represents a very sensitive method to detect and subclassify even lesions that remain ophthalmoscopically invisible [21, 22]. An OCT-based classifier may discern two to three subvisible lesion classes and three to four visible lesion classes as we have shown previously [23].

The present study applies the OCT-based classifier in order to anticipate the area of retina that will ultimately be destroyed by a lesion. Photocoagulation efficacy depends on the totally coagulated area [24], and increased numbers of softer lesions are required for the same clinical effect [14]. Reduced intensity of every single lesion, in order to reduce side-effects like scotoma formation or pain, is invariably accompanied by a reduction of the treated area of retina. A suggested algorithm to calculate the affected retinal area for different intensity lesions used an ophthalmoscopic lesion classifier (moderate, light, barely visible), which is observer- and time-dependent [24]. Therefore, this study examines parameters which are on one hand detectable during or shortly after treatment, more reliable, and more sensitive than ophthalmoscopic lesion evaluation and which are on the other hand correlated with the long-term lesion size.

## 2. Methods

### 2.1. Clinical Study

Photocoagulation lesions were examined in a noninterventional, prospective clinical trial on 20 patients receiving photocoagulation for retinal vein occlusion (3/20), occlusive vasculitis (1/20), and diabetic retinopathy (16/20). Some of the latter were additionally treated focally for diabetic maculopathy (4/16). The study was reviewed and approved by the Institutional Ethics Committee at the University of Kiel (application no. A 105/10) and was carried out in accordance with the contents of the declaration of Helsinki. All treatment indications followed the treatment guidelines of the German ophthalmological society [25, 26].

We chose a study area of 15° × 20° of untreated peripheral retina. This area was imaged by colour fundus images (Zeiss FF450 plus fundus camera, Carl Zeiss Meditec AG, Jena, Germany), AF, infrared (IR), and spectral domain—OCT images (HRA + OCT Spectralis, Heidelberg Engineering, Heidelberg, Germany) before treatment and one hour, one month, three months and six months thereafter. All study lesions were placed within that area. OCT scans of the study area were acquired in 30 *μ*m steps and averaged from 20 individual sweeps. Using the follow-up function (AutoRescan), we traced the lesions through all consecutive OCT series.

We used spot diameters of 100 or 300 *μ*m and exposure-times of 20, 50, or 200 ms. Threshold powers were titrated outside the study area. As the visibility threshold of 100 *μ*m lesions was exceeded with 200 ms exposure-time even at the lowest possible power setting of the photocoagulator (50 mW), we added an additional group of 100 *μ*m, 100 ms lesions. In the study area, we applied rows of five lesions, starting at threshold power and increasing power lesionwise in step widths as provided by the laser device, a modified Zeiss VISULAS VITE 532 nm* continuous wave* laser (50–200 mW: 10 mW-steps, 200–500 mW: 20 mW-steps, > 500 mW: 50 mW-steps). In separate rows of lesions, power was decreased from the threshold in the same manner. Each patient received 20–50 study lesions. 562 study lesions were applied altogether, and 488 of these fell into the areas scanned by OCT. Outside the study area, patients received photocoagulation therapy according to the guidelines.

### 2.2. OCT Cross-Sectional Evaluation

All lesions that could be identified in OCT were mounted in a composite of five images (pretreatment, one hour, one month, three months, and six months). These were arranged in groups with common diameter—exposure-time settings. Within each group, we looked for morphological attributes and ordered the lesions in subgroups with increasing intensity. This led to six consecutive and universal classes of detectable OCT morphologies, irrespective of exposure-time and diameter, as published before [23] and reviewed in Figure 2, top lines.

### 2.3. Photocoagulation Lesion Size Measurements

The greatest linear diameters (GLD) of the lesions were measured in the OCT software. Measurements were carried out in the 1 *μ*m : 1 *μ*m depiction, which we scaled up to 800% magnification. We measured the lesion size at the level of photoreceptor inner segments (IS) or, in class 2 lesions, at the outer nuclear layer (ONL).

Lesion areas were assessed in colour fundus images taken 1 hour after the end of the treatment and IR images taken 1, 3, and 6 months after the treatment. All lesions were contoured manually in image editing software (Gimp 2). All marked lesions' pixel sizes were semiautomatically measured by ImageJ software, and the pixel and real areas were calculated. The scaling factors were retrieved from the camera and OCT manufacturers' softwares. Every lesion was measured by three independent observers. A lesion was considered visible if at least two observers recognized it, and the mean diameter was used for evaluation, but out-of-range values were excluded as described elsewhere [17].

In fundus colour images, we included the bright necrotic lesion core and the greyish denaturation zone into the measurements. An additional halo, which may develop around intense burns, was considered a secondary effect and excluded from the measurements, because it is strongly time-dependent, and exclusion of these halos results in diameter measurements equal to histological damage diameters [27, 28]. In IR images, all reflectivity alterations—bright or dark—that differed from the pretreatment image were included in the lesions size measurement. IR images were evaluated by three independent observers as well.

### 2.4. Statistics

The analyses of the development of GLD and lesion area for different time points were performed by an ANOVA with repeated measurements with and without interaction. Influence variables were observation time (within subject) and exposure-time and irradiation diameter and OCT class (between subjects). Lesions with no measurable GLD or area values (0-values), respectively, were excluded from these analyses.

All performed tests were two-sided. *P* values below 0.05 were considered statistically significant. All statistical analyses were carried out with SPSS software, version 20.

## 3. Results

### 3.1. Clinical Evaluation of Study Lesions (Figure 1)

**Figure 1:**
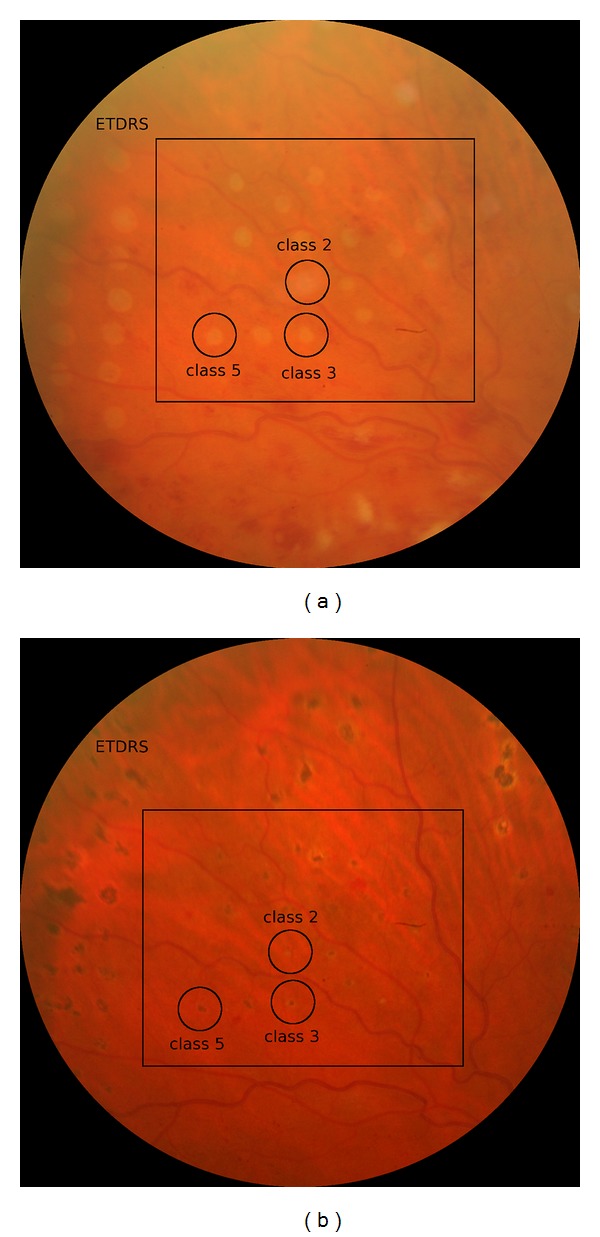
(a) and (b) show colour fundus images of the same study eye taken one hour after the treatment (a) and 6 months after the treatment (b). Examples of study lesions are marked for OCT classes 2, 3, and 5 lesions. The same lesions are displayed in Figures 2 and 3, classes 2, 3, and 5. The class 2 lesion is invisible after 1 hour, but an optical reflex blurs the location. The classes 3 and 5 lesions are visible in (a) and (b). In the periphery of the fundus, we placed standard panretinal lesions (300 *μ*m, 30 ms, moderate grey).

Figure 1 shows fundus images of a typical study area of retina, which contains 4 rows with 5 lesions each, not all of which are visible. Compared to the standard panretinal lesions (300 *μ*m, 30 ms) in the periphery, study lesions were soft, as is demonstrated in Figure 1 at the examples of classes 2, 3, and 5 lesions. The difference is already detectable in 1 hour colour fundus images but becomes more obvious in the 6 months image.

### 3.2. OCT Classification and Qualitative Analysis (Figure 2)

**Figure 2:**
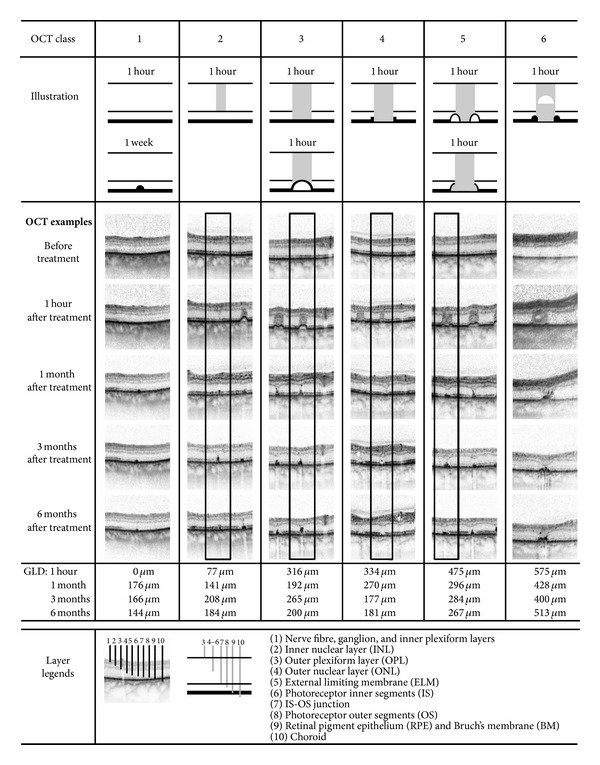
showing representative lesions for 6 consecutive, OCT-based damage classes as defined in a former study [23]. Illustrations of each class are shown at the top of the columns and below representative OCT images taken before the treatment and 1 hour, 1 month, 3 months, and 6 months after the treatment. Classes 3 and 5 may have different OCT appearances, depending on irradiation diameter and exposure-time. All images in a column show the same fundus lesion during follow-up. If an OCT image series shows more than one lesion, a black box demarcates the lesion of interest. At the bottom, the corresponding greatest linear diameters (GLD) are given for all time points, and below that the legend and abbreviations are defined. Physical parameters and clinical images of the same set of lesions are shown in Figure 3.

Figure 2 reviews characteristics of the OCT lesion classification in the top rows. The definition of those previously published OCT lesion classes [23] is summarized as follows. Class zero is undetectable. Class one is invisible one hour post-treatment, but detectable at least in OCT images after 1 week. Class two is barely visible in the outer nuclear layer (ONL) after one hour, and class three is clearly visible with an inner segment (IS)—outer segment (OS) junction line interruption after one hour. Class four shows a thinned RPE/Bruch's membrane (BM) complex and RPE elevations at the lesion border. Class five has thinned RPE in the centre and is surrounded by a ring of detached or excavated RPE. Class six has a bright spot in the centre of the dark column in the ONL. The OCT classifier is useful to discern photocoagulation intensity objectively and very accurately.

The lower lines of the composite in Figure 2 show how lesions developed over 6 months in OCT images and give the OCT GLD measurements for each example at all 4 posttreatment time points. We imaged 488 lesions total with OCT. The qualitative OCT analysis of lesion morphologies after one, three, and six months showed that the extent of retinal damage, which includes axial and horizontal extension of OCT alteration, increases with increasing classes and tends to decrease over time.

### 3.3. Qualitative Analysis of Clinical Lesion Appearance (Figure 3)

**Figure 3:**
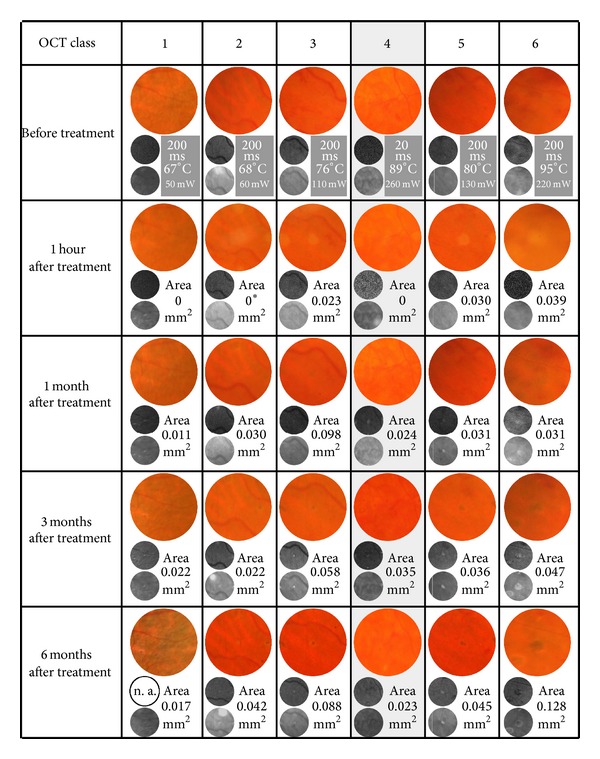
showing the clinical appearance of the lesions from Figure 2 at identical time points (before the treatment, 1 hour, 1 month, and 3 and 6 months after the treatment). Columns compare images of the same lesion over time, and rows compare lesions of different OCT classes at identical time points. Every cell in the composite figure contains a fundus colour image (top), an AF image (middle), and an IR image (bottom). The lesions are centred in all single images. In the top row, we show for each lesion the parameters exposure-time in ms, peak end temperature at the level of the RPE in °C, and power in mW. In the second row (1 hour), the lesion area as measured in the colour image is given in mm², and in all consecutive rows (1–6 months), the lesion area as measured in IR images is given in mm². The irradiated area of 300 *μ*m lesions is 0.071 mm². *In the 1 hour class 2 fundus colour image, the lesion site is superimposed by an optical reflex, but the lesion itself is ophthalmoscopically invisible (see also Figure 1). n. a.: image not available.

Figure 3 complements Figure 2 by showing additional data (power, exposure-time, and peak end temperature) and clinical images for the same lesions and at the same time points (fundus colour images and AF and IR images) as Figure 2. Damage areas as measured in colour images (1 hour) or IR images (1–6 months) are indicated as well. Increasing lesion classes show increasing damage areas.

Very mild lesions become apparent in fundus colour images after one hour but are undetectable in the corresponding IR or AF images. Later, mild lesions may vice versa be detectable only in IR and AF images. The comparison of IR and AF images reveals that both show exactly the same discoloration pattern and are, in this clinical context, equivalent. Due to better image quality and availability in some patients, we chose to evaluate IR images to assess lesion areas in this study.

### 3.4. Sensitivity of Lesion Detection in Fundus Colour (1 Hour), OCT (1 Month), and IR Images (6 Months, Figure 4)

**Figure 4:**
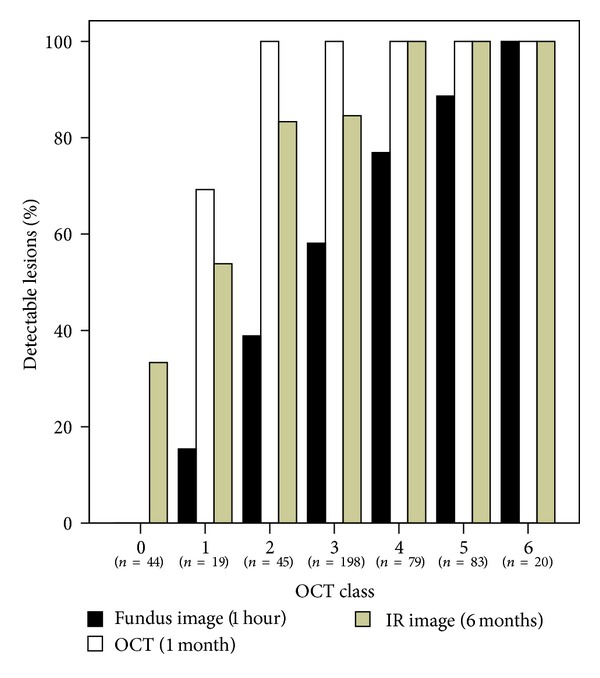
showing percentages of lesion visibilities for the different OCT classes in different examinations. All of OCT class 1 lesions were by definition visible in OCT images after 1 week. Several of these very mild lesions were no longer detectable after 1 month, which explains that less than 100% were detected. Sample sizes for each group are given below the *X*-axis.

Figure 4 shows percentages of lesion visibilities for the different OCT classes in different examination methods. Classes 1 and 2 lesions were rarely ophthalmoscopically visible after 1 hour. There is a clear correlation of lesion class and visibility. The OCT image after 1 month is the most sensitive, followed by IR (6 months) and fundus images (1 hour). Notably, about 1/3 of class 0 lesions, that could be detected neither on the fundus image nor in OCT, caused an altered IR reflectance after 6 months.

Immediate visibility rates during treatment were lower than after 1 hour, as whitening increases over hours after lesion application. Compared to clinical treatment conditions, our detection sensitivity was optimized due to digital upscaling, contrast enhancement, and threefold evaluation by independent investigators. Consequently, lesion classes 1, 2, and possibly 3 would have been considered subvisible in clinical routine evaluation during the treatment. All classes 1 and 2 lesions became, by definition, detectable in OCT images, which gives evidence that they do induce structural retinal damage.

### 3.5. OCT GLD over Time (Figure 5)

**Figure 5:**
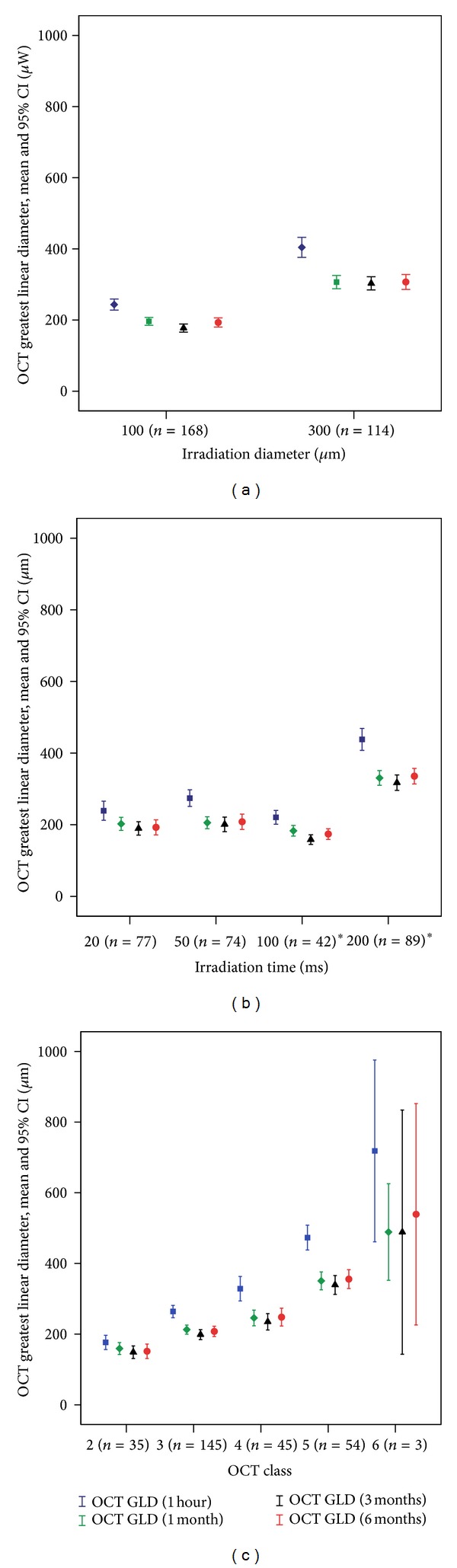
(a)–(c) show OCT GLD from 1 hour to 6 months after treatment. The symbols indicate mean values, and error bars indicate 95% confidence intervals of the mean (CI). In (a), values are grouped in strata of different irradiation diameters, in (b), in strata of different exposure-times, and, in (c), in strata of different OCT classes. *Y*-axes are commonly scaled in all 3 graphs. Sample sizes are indicated at the *X*-axis. *Please note that, in (b), 100 ms lesions were all applied with 100 *μ*m irradiation diameter, and 200 ms lesions, mostly with 300 *μ*m diameter, which leads to a bias of GLD.

Figure 5 shows the greatest linear diameters (GLD) as measured in OCT at the time points 1 hour, 1 month, 3 months, and 6 months after the treatment. The corresponding mean values are given numerically in the supplementary table in the Supplementary Material available online at http://dx.doi.org/10.1155/2014/492679. Stratification according to irradiation diameter is shown in Figure 5(a), according to exposure-time in Figure 5(b), and according to OCT class in Figure 5(c). Lesions that were not detectable in OCT images (GLD = 0 at any time point) were excluded from the analysis. Consequently, class 1 lesions are not displayed.

The greatest change of GLD was a relevant decrease from 1 hour to 1 month after the treatment. There were only small changes after 1 month. From month 3 to 6, slight growth may be suspected in stronger lesions.

The influence of all three examined factors (irradiation diameter, irradiation time, and OCT class) on the GLD is significant (*P* < 0.001), but OCT classes create the greatest bandwidth of GLD's after 1 hour (177 *μ*m in class 2 to 717 *μ*m in class 6), compared to diameter strata (243 *μ*m in 100 *μ*m lesions to 404 *μ*m in 300 *μ*m lesions) and exposure-time strata (239 *μ*m in 20 ms exposures to 438 *μ*m in 200 ms exposures).

The statistical interaction was significant for GLD changes and irradiation diameter (*P* < 0.001) and GLD changes and OCT class (*P* < 0.001), but not for GLD changes and irradiation time (*P* = 0.054). This indicates that GLD changes similarly over time irrespective of irradiation time, but differently for different irradiation diameters or OCT classes. The statistical evaluation of IR lesion area development over time gave similar results (not shown).

Notice that, in Figure 5(b), the 100 ms lesions were all applied at 100 *μ*m irradiation diameter, which produces a bias toward smaller GLD values. The vast majority of 200 ms lesions, in contrast, were applied at 300 *μ*m irradiation diameter (192/234), which produces a bias toward larger GLD values.

### 3.6. Power: Intensity Correlation (Figure 6)

**Figure 6:**
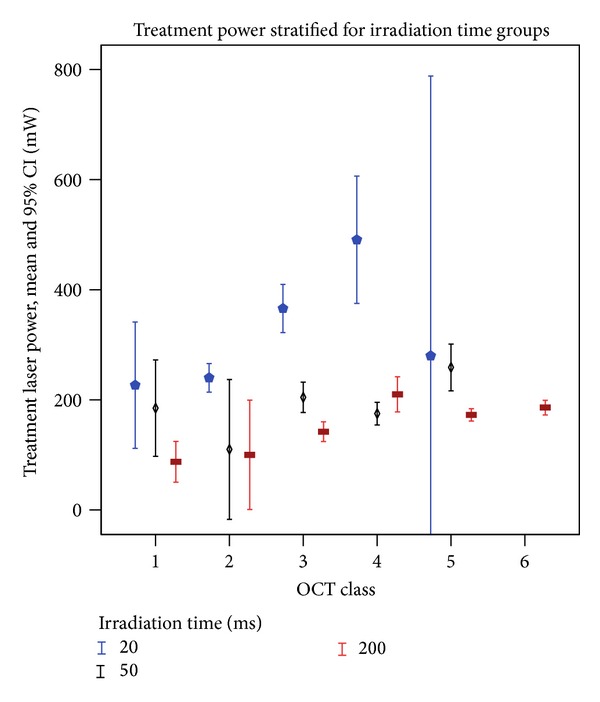
showing laser powers that achieved different OCT classes. Symbols indicate mean values, and error bars indicate CI. Class 5 lesions were rarely achieved by 20 ms exposures, and the low sample size of only 2 lesions limits the validity of the subgroup data shown.

Figure 6 shows laser powers that achieved different OCT classes, and data are stratified for exposure-times. As expected by clinical experience and theoretically described by the Arrhenius theory [29], longer exposures require lower power to achieve a given damage intensity or OCT class, respectively. This correlation is best appreciated in the class 3 data set. For increasing classes at a given exposure-time, increasing mean power values are expected and indeed found for 20 ms, classes 1–4 lesions. In other groups, such as 200 ms, classes 4–6 lesions, we did not encounter this correlation. Low lesion classes 1 and 2 and all 20 ms lesions have large confidence intervals, which indicates that a given power setting may create highly variable lesions in these subgroups, and that these subgroups cannot be reliably controlled by conventional power control. The data show the limitation of power-dependent retrospective laser control and give evidence of the high impact of transmission and pigmentation variation, which accounts for variable effects of lesions applied with identical power. Temperature data of the same set of lesions, which we have published before [23], underline that the inaccuracy is indeed owed to effect variation at constant powers, not to intensity assessment inaccuracy.

## 4. Discussion

In this study we evaluated the laser-induced retinal changes of 488 CW photocoagulation lesions from 20 patients. We varied exposure-time, irradiation diameter, and lesion intensity systematically and observed the lesions over 6 months. Our parameter variations included the clinically most important sets, with exposure-times of 20–200 ms and irradiation diameters of 100 and 300 *μ*m. As influence variables, we examined exposure-time, irradiation diameter, OCT lesion class, and treatment laser power. Outcome measures were obtained in ophthalmoscopy and OCT and IR images and included lesion visibility, diameter, and area. The study addressed the questions which imaging modality was most sensitive and which early parameter was most suitable to estimate the retinal defect size after 6 months.

OCT has been used to display photocoagulation lesions as early as 1995, when Toth et al. applied OCT for a histological correlation of photocoagulation lesions [30]. Detection of subthreshold photocoagulation lesions by higher quality spectral domain OCT has been introduced in 2008 [31] and repeatedly published since then [21, 23, 32, 33]. It has been shown that OCT is capable of detecting lesions that are ophthalmoscopically invisible, and that lesions may be invisible in early OCT images but appear later in follow-up OCT images. OCT has also been used in animal experiments to monitor photocoagulation in real time [34, 35]. Since OCT changes appear with a temporal delay, as do ophthalmoscopic changes, the applicability of OCT might be limited for real-time laser control. In our systematic comparison of mild and subthreshold photocoagulation lesions, IR imaging after 6 months was very sensitive and detected changes in one third of lesions that never became visible in OCT images. OCT images were more sensitive than ophthalmoscopy after 1 hour. Among OCT images, sensitivity after 1 week [23] or 1 month was highest and facilitated objective classification of lesions.

We graded lesions' intensities according to a morphological OCT classifier [23], which is universally applicable to lesions with different irradiation diameters and irradiation times. It was developed with 532 nm CW lesions but would most likely be applicable to differently created lesions as well, which is confirmed by findings of Mojana et al. who observed some similar OCT changes in “subthreshold” lesions created with an IR micropulse laser [21]. The OCT classifier includes two different subvisible intensity grades, which were in this case created with a CW photocoagulator. We have proven the validity of the OCT classifier on short-term follow-up data before [23]. In the present study, we show that lesion intensity is the key measure to describe the tissue effect of photocoagulation, as its predictive value concerning the 6-month lesion GLD (152–539 *μ*m) and area is much better than that of power (Figure 6), irradiation diameter, or irradiation time (193–336 *μ*m, Figure 5). OCT lesion intensity classification includes the impact of local and individual transmission and pigmentation as well. The presented lesion assessment has the potential to define common treatment endpoints that would be universally applicable to different photocoagulators (CW versus pulsed and different wavelengths) and protocols (irradiation times and duty cycles).

Numerous studies have investigated photocoagulation lesions after clinical treatment protocols, partly with long clinical follow-up, but with low parameter variation [19, 21, 22, 32, 36–40]. Mojana et al. evaluated subvisible IR-laser lesions in OCT images and discriminated 3 different lesion classes, some of which were comparable to ours, but which did not cover the entire intensity range [21]. Fewer OCT studies varied lesion parameters systematically [19, 20, 24, 41]. The lesion intensities displayed in those studies match our classes 3 (barely visible in [24]/subvisible in [20, 41]) to 6 (moderate grade in [24]/suprathreshold in [20, 41]), while our 2012 publication was the first systematic investigation that described subthreshold classes 1 and 2 [23].

Muqit et al. investigated 120 photocoagulation lesions of 392 *μ*m diameter after 20, 100, or 200 ms irradiations at four different ophthalmoscopic endpoints. They observed decreasing GLD particularly for shorter exposed or less intense lesions in direct comparison of 1 hour and 6 month OCT images [41]. Lavinsky et al. analysed 100–400 *μ*m, 10–200 ms lesions in OCT images over one year [20]. These authors emphasize the advantages of shortly exposed, smaller lesions when controlled by ophthalmoscopic visibility. In contrast, we believe that microstructural lesion intensity is the key measure which is correlated with clinical efficacy and biological response to laser irradiation. In fact, the lack of interaction of exposure-time and GLD development over time gives a clue that exposure-time might not be an adequate measure to differentiate lesions with different growth behaviour.

Photocoagulation scars grow frequently. Reported incidences range from 5.4% of eyes after mild macular treatment [42] to 70% of lesions after strong subretinal neovascularization treatment. Growth occurred at any time point after the treatment, resulted in geographic retinochoroidal atrophy [4], and can be detrimental in macular lesions [42]. On the other hand, complete retinal restoration after mild photocoagulation has been described and controversially discussed as well [19–22, 43, 44]. In our study, we did not encounter complete lesion disappearance in OCT images except for 5/488 (1%) lesions, in spite of the lowest possible lesion intensity. Concerning growth, obviously either the 6-month time frame of this study was too short to see significant growth or the lesions were too mild to grow substantially.

Lavinsky et al. have previously shown that short exposure or mild panretinal treatment affects a smaller area of retina if compared to standard panretinal treatment, and that there is an inverse correlation of the totally affected retinal area and clinical effectiveness [14, 24]. The lesion they show in [24] as an example of standard ETDRS treatment would be class 6 according to our classification (final GLD 539 *μ*m). In our study, the classes 3 and 4 final GLD was 225 *μ*m, which is 42% of the class 6 diameter. Hence, the treated area of classes 3-4 lesions is about 18% (0.42²) of class 6 lesions. The final GLD of class 2 lesions was 150 *μ*m, which is 28% of the class 6 diameter, and their treated area is about 8% (0.28²) of class 6 lesions. In order to treat the same retinal area, about 5 times as many class 3 or 4 lesions and 12 times as many class 1 or 2 lesions would be necessary.

The physical laser parameters are only loosely correlated with the intensity of a retinal lesion, and even the ophthalmoscopic lesion appearance gives limited information on how severe a lesion is on a cellular level. Figure 3 demonstrates the strong ophthalmoscopic similarity of different OCT-class lesions. In our previous study, we examined 35 lesions that had been applied with 100 *μ*m diameter and 100 ms exposure-time in mild macular treatment, and of these, 12 (34%) were class 3 and 23 (66%) were class 4 [23]. 300 *μ*m, 200 ms lesions were mostly class 5 (59%) but included some class 6 (31%) and few classes 3 and 4 lesions (each 5%) as well, although all lesions aimed at a common ophthalmoscopic endpoint. An ongoing, unpublished study shows that lesion intensities are highly variable within the same treatment session and between different physicians and different patients treated by the same physician. Obviously, ophthalmoscopic lesion evaluation is not very reliable.

## 5. Conclusions

Therefore, assessment of OCT lesion classes as investigated in this study would, for the first time, facilitate comparability of differently created photocoagulation lesions across different studies. It would also allow a predictive estimation of the clinical effect by the presented correlation with the final lesions sizes and enable calculation of necessary lesion numbers on the basis of a much more reliable measure than ophthalmoscopic lesion class.

## Supplementary Material

Supplementary Table: indicates OCT GLD after treatment numerically, indicating in 4 columns the values 1 hour, 1, 3 and 6 months after treatment. The same data are shown graphically in Fig. 5 a) - c), where we also give sample sizes. In the upper rows, values are shown for strata of different irradiation diameters, in the middle rows, for different exposure times, and in the lower rows, for different OCT classes, respectively. Any data set that contained a 0-value was excluded from the evaluation.
*In these groups, irradiation times were unevenly distributed, which induces a bias."

## References

[1] Shah AM, Bressler NM, Jampol LM (2011). Does laser still have a role in the management of retinal vascular and neovascular diseases?. *American Journal of Ophthalmology*.

[2] Mainster MA (1999). Decreasing retinal photocoagulation damage: principles and techniques. *Seminars in Ophthalmology*.

[3] Fong DS, Girach A, Boney A (2007). Visual side effects of successful scatter laser photocoagulation surgery for proliferative diabetic retinopathy: A Literature Review. *Retina*.

[4] Morgan CM, Schatz H (1989). Atrophic creep of the retinal pigment epithelium after focal macular photocoagulation. *Ophthalmology*.

[5] Lanzetta P, Dorin G, Pirracchio A, Bandello F (2001). Theoretical bases of non-ophthalmoscopically visible endpoint photocoagulation. *Seminars in Ophthalmology*.

[6] Roider J, Brinkmann R, Wirbelauer C, Laqua H, Birngruber R (1999). Retinal sparing by selective retinal pigment epithelial photocoagulation. *Archives of Ophthalmology*.

[7] Dorin G (2003). Subthreshold and micropulse diode laser photocoagulation. *Seminars in Ophthalmology*.

[8] Paulus YM, Jain A, Nomoto H (2011). Selective retinal therapy with microsecond exposures using a continuous line scanning laser. *Retina*.

[9] Muqit MMK, Gray JCB, Marcellino GR (2010). Barely visible 10-millisecond pascal laser photocoagulation for diabetic macular edema: observations of clinical effect and burn localization. *American Journal of Ophthalmology*.

[10] Roider J, Liew SHM, Klatt C (2010). Selective retina therapy (SRT) for clinically significant diabetic macular edema. *Graefe’s Archive for Clinical and Experimental Ophthalmology*.

[11] Koinzer S, Elsner H, Klatt C (2008). Selective retina therapy (SRT) of chronic subfoveal fluid after surgery of rhegmatogenous retinal detachment: three case reports. *Graefe’s Archive for Clinical and Experimental Ophthalmology*.

[12] Klatt C, Saeger M, Oppermann T (2011). Selective retina therapy for acute central serous chorioretinopathy. *British Journal of Ophthalmology*.

[13] Luttrull JK, Musch DC, Spink CA (2008). Subthreshold diode micropulse panretinal photocoagulation for proliferative diabetic retinopathy. *Eye*.

[14] Lavinsky D, Cardillo JA, Melo LAS, Dare A, Farah ME, Belfort R (2011). Randomized clinical trial evaluating mETDRS versus normal or high-density micropulse photocoagulation for diabetic macular edema. *Investigative Ophthalmology & Visual Science*.

[15] Luttrull JK, Spink CJ (2006). Serial optical coherence tomography of subthreshold diode laser micropulse photocoagulation for diabetic macular edema. *Ophthalmic Surgery Lasers and Imaging*.

[16] Vujosevic S, Bottega E, Casciano M, Pilotto E, Convento E, Midena E (2010). Microperimetry and fundus autofluorescence in diabetic macular edema: subthreshold micropulse diode laser versus modified early treatment diabetic retinopathy study laser photocoagulation. *Retina*.

[17] Koinzer S, Schlott K, Ptaszynski L (2012). Temperature controlled retinal photocoagulation—a step toward automated laser treatment. *Investigative Ophthalmology & Visual Science*.

[18] Figueira J, Khan J, Nunes S (2009). Prospective randomised controlled trial comparing sub-threshold micropulse diode laser photocoagulation and conventional green laser for clinically significant diabetic macular oedema. *British Journal of Ophthalmology*.

[19] Muqit MMK, Gray JCB, Marcellino GR (2010). In vivo laser-tissue interactions and healing responses from 20- vs 100-millisecond pulse pascal photocoagulation burns. *Archives of Ophthalmology*.

[20] Lavinsky D, Cardillo JA, Mandel Y (2013). Restoration of retinal morphology and residual scarring after photocoagulation. *Acta Ophthalmologica*.

[21] Mojana F, Brar M, Cheng L, Bartsch D-UG, Freeman WR (2011). Long-term SD-OCT/SLO imaging of neuroretina and retinal pigment epithelium after subthreshold infrared laser treatment of drusen. *Retina*.

[22] Deák G, Bolz M, Prager S (2012). Photoreceptor layer regeneration is detectable in the human retina imaged by SD OCT after laser treatment using sub-threshold laser power. *Investigative Ophthalmology & Visual Science*.

[23] Koinzer S, Schlott K, Portz L (2012). Correlation of temperature rise and optical coherence tomography characteristics in patient retinal photocoagulation. *Journal of Biophotonics*.

[24] Palanker D, Lavinsky D, Blumenkranz MS, Marcellino G (2011). The impact of pulse duration and burn grade on size of retinal photocoagulation lesion: implications for pattern density. *Retina*.

[25] (2011). Recommendation of the Retinological Society, the German Ophthalmological Society and the Professional Association of Ophthalmologists in Germany: treatment of diabetic maculopathy. *Klinische Monatsblätter für Augenheilkunde*.

[26] (2010). Statement of the German Ophthalmological Society, the Retinological Society and the Professional Association of German Ophthalmologists on Therapy for Macular Oedema in Cases of Retinal Vein Occlusion. *Klinische Monatsblätter für Augenheilkunde*.

[27] Weinberg W, Gabel V-P, Birngruber R (1981). Time sequence of the white hue correlated with the extent of damage in photocoagulation of the retina. *Berichte der Deutschen Ophthalmologischen Gesellschaft*.

[28] Koinzer S, Saeger M, Hesse C (2013). Correlation with OCT and histology of photocoagulation lesions in patients and rabbits. *Acta Ophthalmologica*.

[29] Birngruber R, Hillenkamp F, Gabel V-P (1985). Theoretical investigations of laser thermal retinal injury. *Health Physics*.

[30] Toth C, Birngruber R, Fujimoto J (1995). Correlation between optical coherence tomography, clinical examination and histopathology of macular laser lesions. *Investigative Ophthalmology & Visual Science*.

[31] Lanzetta P, Polito A, Veritti D (2008). Subthreshold Laser. *Ophthalmology*.

[32] Framme C, Walter A, Prahs P (2009). Structural changes of the retina after conventional laser photocoagulation and selective retina treatment (SRT) in spectral domain OCT. *Current Eye Research*.

[33] Deák GG, Bolz M, Prager S (2012). Photoreceptor layer regeneration is detectable in the human retina imaged by SD-OCT after laser treatment using subthreshold laser power. *Investigative Ophthalmology & Visual Science*.

[34] Veritti D, Sarao V, Lanzetta P (2012). Online optical coherence tomography during subthreshold laser irradiation. *European Journal of Ophthalmology*.

[35] Müller HH, Ptaszynski L, Schlott K (2012). Imaging thermal expansion and retinal tissue changes during photocoagulation by high speed OCT. *Biomedical Optics Express*.

[36] Nagpal M, Marlecha S, Nagpal K (2010). Comparison of laser photocoagulation for diabetic retinopathy using 532-nm standard laser versus multispot pattern scan laser. *Retina*.

[37] Kang H, Su L, Zhang H, Li X, Zhang L, Tian F (2010). Early histological alteration of the retina following photocoagulation treatment in diabetic retinopathy as measured by spectral domain optical coherence tomography. *Graefe’s Archive for Clinical and Experimental Ophthalmology*.

[38] Bolz M, Kriechbaum K, Simader C (2010). In vivo retinal morphology after grid laser treatment in diabetic macular edema. *Ophthalmology*.

[39] Kriechbaum K, Bolz M, Deak GG, Prager S, Scholda C, Schmidt-Erfurth U (2010). High-resolution imaging of the human retina in vivo after scatter photocoagulation treatment using a semiautomated laser system. *Ophthalmology*.

[40] Luttrull JK, Sramek C, Palanker D, Spink CJ, Musch DC (2012). Long-term safety, high-resolution imaging, and tissue temperature modeling of subvisible diode micropulse photocoagulation for retinovascular macular edema. *Retina*.

[41] Muqit MMK, Denniss J, Nourrit V (2011). Spatial and spectral imaging of retinal laser photocoagulation burns. *Investigative Ophthalmology and Visual Science*.

[42] Schatz H, Madeira D, McDonald HR, Johnson RN (1991). Progressive enlargement of laser scars following grid laser photocoagulation for diffuse diabetic macular edema. *Archives of Ophthalmology*.

[43] Paulus YM, Jain A, Gariano RF (2008). Healing of retinal photocoagulation lesions. *Investigative Ophthalmology and Visual Science*.

[44] Stanga PE, Muqit MMK (2010). Re: comparison of laser photocoagulation for diabetic retinopathy using 532-nm standard laser versus multispot pattern scan laser. *Retina*.

