# Supplementary material for: Comprehensive Detection, Grading, and Growth Behavior Evaluation of Subthreshold and Low Intensity Photocoagulation Lesions by Optical Coherence Tomographic and Infrared Image Analysis
Source: Biomed Res Int. 2014 May 12;2014:492679. doi: 10.1155/2014/492679 (PMC4037579; doi:10.1155/2014/492679)
Supplement: Supplementary file 1 — Supplementary Table: indicates OCT GLD after treatment numerically, indicating in 4 columns the values 1 hour, 1, 3 and 6 months after treatment. The same data are shown graphically in Fig. 5 a) - c), where we also give sample sizes. In the upper rows, values are shown for strata of different irradiation diameters, in the middle rows, for different exposure times, and in the lower rows, for different OCT classes, respectively. Any data set that contained a 0-value was excluded from the evaluation. *In these groups, irradiation times were unevenly distributed, which induces a bias." [file 492679.f1.pdf]

Tables

Suppl. Tbl. shows mean OCT GLD's at all 4 time points examined for different lesion stratifications. The same data are displayed graphically in Fig. 5. 0-values were excluded from the evaluation.

* In these groups, irradiation diameters were unevenly distributed, which leads to a bias.

Stratification criterion	1 hour OCT GLD [µm]	1 month OCT GLD [µm]	3 months OCT GLD [µm]	6 months OCT GLD [µm]
Diameter 100 µm	243	196	178	193
Diameter 300 µm	404	307	303	307
Time 20 ms	239	202	190	193
Time 50 ms	274	206	201	208
Time 100 ms*	221	183	159	174
Time 200 ms*	439	331	317	336
OCT class 2	177	159	149	152
OCT class 3	264	213	199	208
OCT class 4	329	246	235	248
OCT class 5	473	351	339	356
OCT class 6	717	489	489	539
